# Prevalence and Correlates of Unmet Supportive Needs of Nigerian Patients With Cancer

**DOI:** 10.1200/JGO.19.00043

**Published:** 2019-06-27

**Authors:** Olamijulo Fatiregun, Anthonia Chima Sowunmi, Muhammad Habeebu, Paul Okediji, Adewumi Alabi, Omolara Fatiregun, Adeoluwa Adeniji, Opeyemi Awofeso, Bolanle Adegboyega

**Affiliations:** ^1^ Lagos University Teaching Hospital, Idi-araba, Lagos, Nigeria.; ^2^ University of Lagos, Lagos, Nigeria; ^3^ Solar Center for International Development and Research, Abuja, Nigeria

## Abstract

**PURPOSE:**

Diagnosis and treatment of cancer are associated with significant psychological distress, and patients face a broad range of challenges that create a vacuum of unmet needs felt by patients, such as a loss of personal control and frustration. The aim of the current study was to determine the magnitude, distribution, and correlates of unmet needs in Nigerian patients with cancer.

**PATIENTS AND METHODS:**

Using a descriptive cross-sectional approach, we assessed 205 patients with cancer who attended oncology outpatient clinics at the Lagos University Teaching Hospital. Eligible patients were administered the Supportive Care Needs Survey (SCNS) –Short Form 34 with a focus on five domains of need: psychological, health system and information, physical and daily living, patient care and support, and sexuality.

**RESULTS:**

Mean age was 47.4 ± 12.3 years and patients were predominantly female (96.6%). The most common diagnosis was breast cancer (92.2%), and mean duration since diagnosis was 20.9 ± 21.9 months for all patients. Mean SCNS score was 83.9 ± 24.8 and at least 46% of participants indicated unmet needs in 15 items of the SCNS. The most frequent unattended needs were related to the health information (53.4%), physical and daily living (49.4%), psychological (48.5%), sexuality, and patient care and support domains. None of the factors considered—age, sex marital status, family type, educational attainment, employment status, economic status, the presence of financial support, social support, and cancer type—was significantly predictive of unmet needs in these patients (*P* > .05).

**CONCLUSION:**

Nigerian patients with cancer experience considerable levels of unmet needs. These needs require urgent and long-term interventions to help patients achieve increased care satisfaction and a better quality of life.

## INTRODUCTION

Cancer is a major cause of morbidity and mortality globally, with more than one million new cases diagnosed annually.^1^ In Nigeria, the incidence of cancer has been increasing, with approximately 100,000 new cases coming up each year as a result of increased westernization; increased cancer awareness; reduced mortality from infectious diseases; increasing life expectancy; and changes in sociodemographic, economic, and epidemiologic risk factors.^1,2^ Despite increased advocacy and awareness, however, late presentation for diagnosis and care is the norm as patients wait until the disease enters its later stages before presenting for care.^3^

**CONTEXT**

**Key Objective**
The aim of the current study was to determine the magnitude and distribution of unmet needs in Nigerian patients with cancer using a descriptive cross-sectional approach in a tertiary institution in Lagos, Nigeria. We used the Supportive Care Needs Survey–Short Form 34 with focus on five domains of need: psychological, health system and information, physical and daily living, patient care and support, and sexuality.
**Knowledge Generated**
Of participants, 46% indicated unmet needs in 15 items of the Supportive Care Needs Survey and the most frequently unmet needs were related to health information, psychological, and physical and daily living needs. The most frequently met needs were sexuality and patient care and support needs.
**Relevance**
We concluded that many of our patients with cancer experience significant levels of unmet needs and that these needs require urgent and long-term interventions to help patients achieve increased care satisfaction and a better quality of life.

Diagnosis of cancer has been associated with substantial psychological distress as most patients are caught off-guard and often not prepared to handle the initial mental stress that the diagnosis brings.^4^ As treatment progresses, the patient faces a broad range of financial, emotional, social, psychological, and physical challenges emanating from the disease itself, its perceptions, and its treatment.^5^ Considering the present opinions of cancer being a death sentence in Nigeria, many patients feel despondent and give up hope, believing that they have minimal chances of survival.^6^ Moreover, it is generally accepted that common fears, such as a fear of metastasis, recurrence, pain, and distress from surgery; fear of developing another cancer; and the fear of being unable to cope with the costs of treatment, negatively affects psychological wellbeing.^7^

In addition to the elevated levels of psychological distress faced by patients with cancer, evidence indicates that morbidity associated with the disease reduces the general quality of life of the patient, as it impairs the ability to maintain work activities, home management, family and social relationships, sexual activity, and sleep patterns.^8-10^ These lingering problems translate into a considerable burden of unmet needs, which patients experience as a loss of personal control, frustration, and deficiencies in other areas of life occurring as a result of the primary cancer diagnosis.^11^ On the basis of the understanding that the overall wellbeing of a patient with cancer is important in the treatment of the primary disease, there is increased interest in improving the patient’s psychosocial wellbeing by identifying and satisfying the unmet needs.^12,13^ Efforts to elucidate the most effective ways to satisfy the unattended needs of patients with cancer and increase their quality of life highlight the need for proper assessment and analysis of the relationships between these neglected needs and possible associated factors.

Several studies outside Africa have highlighted the considerable psychosocial burden that patients with cancer experience.^14-17^ As a result of varying results across populations, however, there is currently no consensus around which aspects of a patient’s care—in addition to clinical care—require more attention to provide wholesome care, increase patient satisfaction, and improve treatment outcomes. Similarly, the influence of factors that determine unmet needs vary widely, indicating the need to consider the peculiarities of the index population. With the understanding that no local study has examined the unsatisfied needs of Nigerian patients with cancer, it becomes necessary to assess the magnitude and factors associated with unmet needs among the local population. Furthermore, a proper assessment of deprived needs enables us to identify patients with higher levels of need rapidly and early enough so that they can be targeted first with necessary interventions that can improve their psychosocial wellbeing. The aim of the current study was to provide information on the magnitude and sociodemographic and clinical correlates of specific unmet needs in Nigerian patients with cancer.

## PATIENTS AND METHODS

This study was carried out at the outpatient cancer clinics of Lagos University Teaching Hospital, Idi-Araba, Nigeria, where patients with various types of cancers present for treatment and follow-up. This medical facility is one of the few equipped with a functional linear accelerator for radiotherapeutic intervention in cancer cases and is a major referral center for patients within and outside Lagos, where more than 4,000 new cancer cases are seen each year.

Participants were selected from those who attended the oncology clinic regularly for treatment and follow-up on the basis of the following eligibility criteria: age 18 years and older, diagnosed with cancer for at least 3 months or more, and mentally stable and literate enough to complete the survey questionnaire. Patients who refused to provide informed consent, had a preexisting mental illness, and who were unable to understand the components of the survey despite reasonable interpretations were excluded from the study.

Using a prevalence of 20% (moderate to high needs),^18^ the sample size was derived from the sample size determination formula *n* = *z*2*pq*/*d*2 at a 95% CI and a 5% margin of error.^19^ As the total clinic enrollment was fewer than 10,000 patients, final sample size was determined to be approximately 200 patients after applying a correction factor in addition to a 10% potential rate of incomplete or poor responses.^20^ Once eligibility was determined, potential participants were selected using a consecutive sampling method by which every eligible patient who attended the clinic was approached for participation. A summary of the purpose of this study was given to each of these patients and informed consent was obtained, after which they were eligible to answers the questionnaire.

### Measures

#### Sociodemographic and clinical questionnaire.

Participants were administered questionnaires aimed at gathering information about their age, marital status, level of education, occupation, and economic status. Clinical data were obtained by reviewing the patient’s hospital folder with a specific focus on cancer diagnosis, duration of illness, family history of cancer, type of treatment(s), and treatment compliance.

#### Supportive Care Needs Survey–Short Form 34.

This short form of the Supportive Care Needs Survey (SCNF) has been used in a wide variety of studies to assess the unmet needs of patients with cancer.^21,22^ It contains 34 items spread across five domains of need: psychological (10 items), health system and information (11 items), physical and daily living (five items), patient care and support (five items), and sexuality (three items).^23^ Using this questionnaire, patients were asked to indicate the level of need for help on the basis of the various aspects of the questionnaire. Possible scores ranged from 34 to 170, and higher scores point at greater perceived needs.

### Data Analysis

To highlight the level and distribution of deprived needs among the sample population, we performed data analysis using Statistical Package for Social Sciences software for Windows (version 19; SPSS, Chicago, IL). Demographic, social, and clinical characteristics of patients, as well as the levels and distribution of deprived needs, were analyzed using descriptive statistics and presented in the form of frequencies, percentages, means, and standard deviation.

### Ethical Considerations

Ethical approval was sought from the ethics committee of the Lagos University Teaching Hospital, and the study was conducted according to the principles of the Declaration of Helsinki. Consideration was made for data confidentiality, nonmaleficence, and beneficence. Informed consent was sought from every participant before undertaking to participate in the study.

## RESULTS

A total of 205 participants were sampled, the majority of them female (96.6%) and married (78.0%; Table 1). More participants had at least a tertiary education (44.9%) and up to 56.6% were unemployed. Most had a moderate economic status (60.9%) and lived with a spouse and/or children (79.5%). Support for taking care of participants’ illnesses came mostly from relatives (70.7%), followed by self-sponsorship (21.9%).

**TABLE 1:**
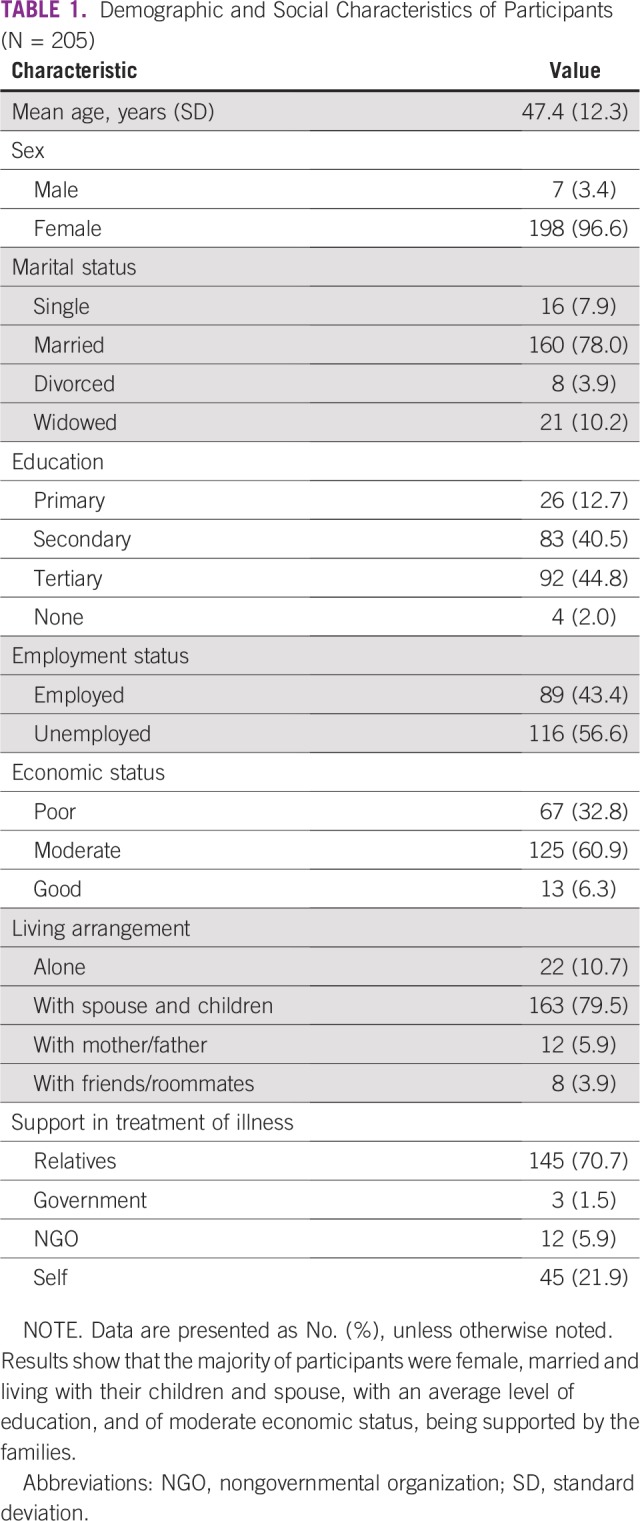
Demographic and Social Characteristics of Participants (N = 205)

The majority of participants sampled suffered from breast cancer (92.2%). Other cancers identified include sarcoma (3.9%), nasopharyngeal carcinoma (1.5%), colorectal cancer, and prostate cancer (1.0%; Table 2). A few patients had a family history of the index illness (9.3%) and up to 32.7% had had a previous history of the disease. Most treatments were in the form of chemotherapy (62.4%), followed by radiotherapy (21.5%) and surgery (19.0%).

**TABLE 2:**
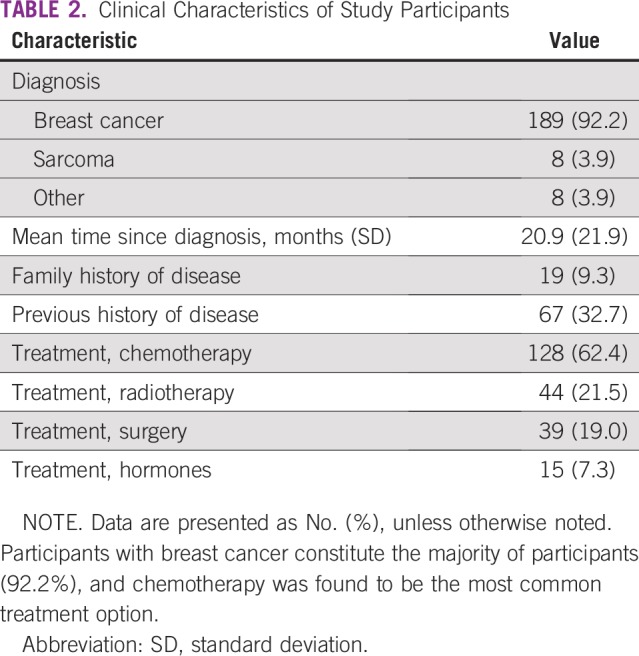
Clinical Characteristics of Study Participants

More than one half of participants experienced at least one unmet need (Table 3); the most frequent unsatisfied needs were in the health system and information domain. At least 51% of participants had concerns with getting information about important aspects of their care, were not informed about what they needed to do to help themselves get well, or whether their cancer is under control or in remission. Items in the psychological domain were also common, as participants expressed that their fears about the spread of cancer, worries of those close to them, anxiety, and how to feel in control of their situation were not addressed.

**TABLE 3:**
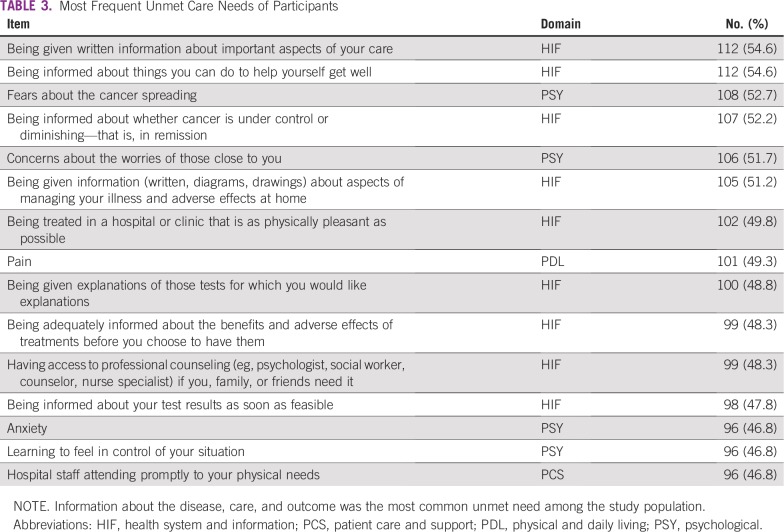
Most Frequent Unmet Care Needs of Participants

Scores across domains are summarized in Table 4. The most unattended needs were in the health system and information domain. On a scale of 1 to 100, the health system and information domain had a score higher than 50. Although scores in the other four areas were lower than 50, the margin was minimal—physical and daily living (49.4), psychological (48.5), patient care and support (47.6), and sexuality needs (44.4).

**TABLE 4:**
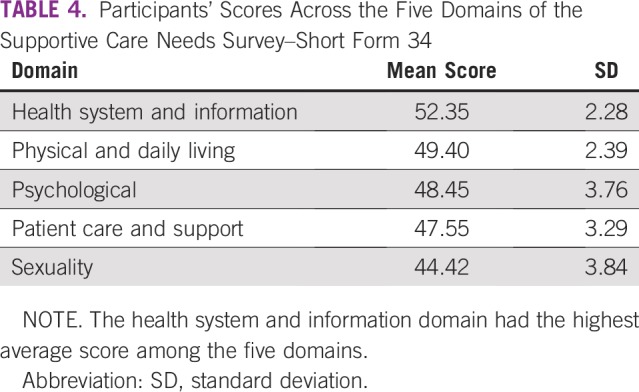
Participants’ Scores Across the Five Domains of the Supportive Care Needs Survey–Short Form 34

Several participants indicated that their needs were met across some items. The most frequent met need was about changes in sexual relationships (69.3%). “Feelings about death and dying” and “Changes in sexual feelings” were also reported to be frequently addressed. Across the top five most met needs (Table 5), items in the sexuality needs domain were prominent. Items in the patient care and support domain and the psychological domain were also frequent, indicating addressed needs in these areas. It seems that participants had more choices of hospitals to which they could go for care and cancer specialists to attend to them (at least 62% each).

**TABLE 5:**
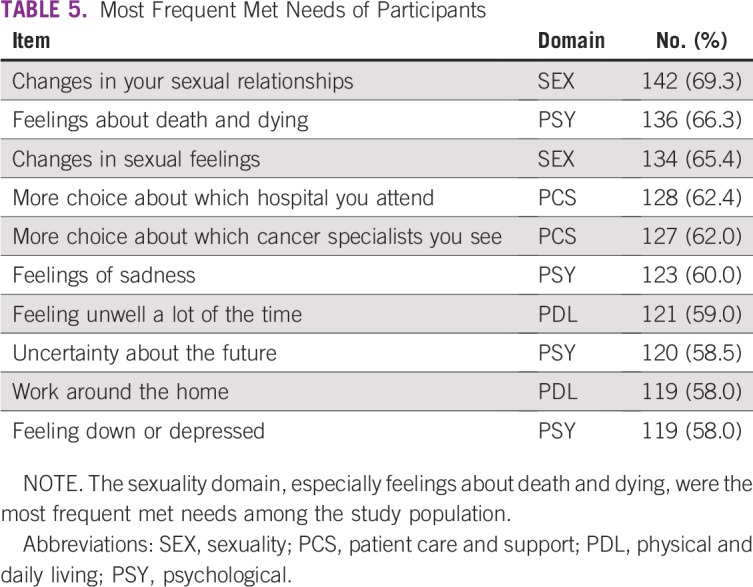
Most Frequent Met Needs of Participants

Mean supportive care scores across domains were compared with specific sociodemographic and clinical characteristics (Table 6). Respondents older than 60 years indicated a significantly lower unmet need in the psychological domain (*P* = .045); however, across the other domains, older participants had more unattended needs—sexuality, patient care and support, and health information—than those who were age 60 years or younger.

**TABLE 6:**
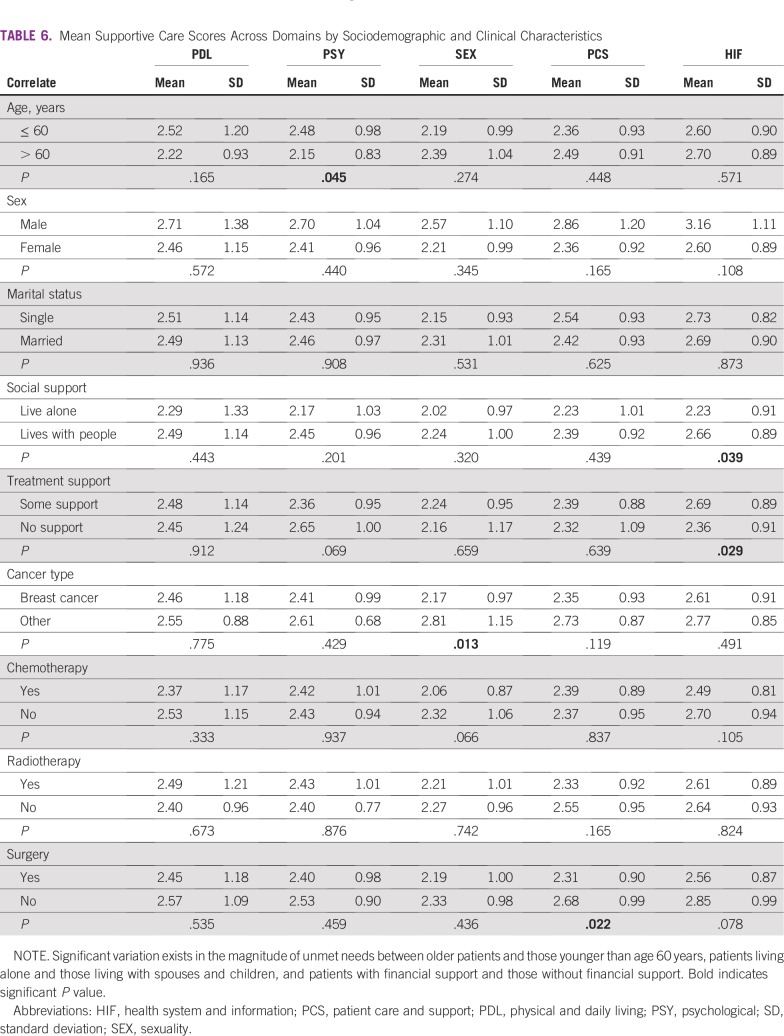
Mean Supportive Care Scores Across Domains by Sociodemographic and Clinical Characteristics

Respondents who lived with other people—parents, spouses, and/or children—reported a significantly higher level of neglected needs in the area of health information (*P* = .039; Table 6) than did those who lived alone. Similarly, participants who received financial support for their care—from any source, including family, friends, or charity—had a higher unmet need for health information (*P* = .029) than did those who do not receive any financial support. Participants with other types of cancer apart from breast cancer had a significantly higher unmet need in the sexuality domain (*P* = .13) than did those with breast cancer. Gender and marital status did not seem to have an impact on the reporting of unmet needs.

## DISCUSSION

This study provides rare insight into the perceptions of patients with cancer concerning deficiencies in their care. It demonstrates that many Nigerian patients with cancer experience unmet needs in various aspects of their care. As shown in Table 3, approximately one half of participants had unfulfilled needs in 15 items of the SCNS-Short Form 34, particularly in the health system and information and psychological domains. This finding is comparable with results obtained in Iran^24^ and Australia,^22^ which suggests a similarity in the increased focus on clinical aspects of care in many cancer treatment centers across the world and less on health information and psychological support.^11,22^

In addition, a key finding in this study is the substantial gap in the area of health information for sampled participants. A number of unfulfilled needs that were expressed by participants were in the health system and information domain. A recent systematic review of studies that were carried out on varying populations reported that the majority of patients with cancer have more unattended needs in the health information domain than in any other domain.^11^ This finding may be a result of the increasing number of patients with cancer that oncologists see, which leads to short consultation times and not enough time with which to address all the questions a patient with cancer might have.^25^ There is also the risk of overwhelming patients with a large amount of information provided by oncologists within a short period of time.^26^

The consistency of this finding across many populations, including the sampled population, indicates the importance of paying more attention to the health information needs of patients. As seen in Table 3, Nigerian patients with cancer are interested in receiving information about the important aspects of their care and what they can do to help themselves get better. Patients also want to be informed about whether their disease is under control or in remission. To combat the information gap, the use of written information, such as diagrams, pictures, and drawings about various aspects of their illness, may be of benefit. The importance of this is reflected in participants’ response to this specific item in the SCNS-Short Form 34, as well as in a study by Moerenhout et al,^27^ which indicated that the provision of patient health information materials in waiting rooms might help optimize patient–physician interaction, as well as health-related knowledge and self-management.

In addition to the gap in health information, many patients indicated unmet needs in the psychological domain. Concerns about the cancer spreading, worries, and anxiety in themselves and those closest to them, as well as being able to feel in control of their situations were paramount among unmet needs experienced by participants. Previous studies have indicated that unmet needs in the psychological domain were widespread among patients with cancer^28,29^ and have contributed, in part, to the advent of a new field of cancer care, psycho-oncology.^30,31^ It is expected that sustained focus on the psychological needs of patients with cancer will translate to fewer unfulfilled needs in this domain.

The most met needs were in the sexuality domain. This is in agreement with results in other studies, particularly in Malaysia, Iran, and other conservative societies like Nigeria.^13,28,29,32,33^ Many conservative organizations discourage an open discussion of sexuality.^11^ There is also some stigma associated with not being able to perform sexual functions appropriately, which may result in false responses to questions about sexuality.^29,34^ It is acceptable to assume that sexual needs are met, but another possibility is that patients may not consider sexual needs to be paramount compared with needs in other areas, such as the psychological, health information, or physical and daily living domains. However, there is evidence linking the sexuality and psychological domains, and a reduced sexual capacity may translate to impaired mental functioning and vice versa.^35,36^

With regard to the correlates of unmet needs in this study, the influence of age was felt only in the psychological domain. Younger participants seemed to have more psychological unfulfilled needs. This finding is similar to that reported by Schmid-Buchi et al^37^ and Griesser et al,^38^ who noted that younger female patients have more needs in this domain; however, gender was not found to have any statistically significant influence on the magnitude of unmet needs across the areas of this study. Patients who received social support seemed to have more of their needs go unmet in the health information domain. It is possible that living with other people stimulated the desire to ask questions and request more information. This finding is tied closely to the fact that study participants who received financial support for their care from other sources—family, friends, or charity—also had a statistically significant increased unmet need for health information. Boyes et al^39^ found that low levels of social support were associated with higher levels of unmet need. Although this study did not measure the level of social support, it indicates that having some level of support may trigger increased levels of need across domains, especially in the health information domain.

Furthermore, patients with other types of cancer, predominantly sarcomas and prostate cancer, had statistically significant higher levels of unmet need in the sexuality domain. Whereas this finding is correlated with the idea that the needs of patients with breast cancer are often different from patients with other types of cancer, it does not point to a reason for the increased need in the sexuality domain. In concluding this finding, however, it is essential to bear in mind the conservative nature of the Nigerian society, which may influence responses to questions in this domain.

The findings in the current study have important implications for the care of patients with cancer. First, it provides evidence showing that many Nigerian patients with cancer experience varying degrees of unsatisfied needs across domains. Second, it establishes a major gap in health information and psychological care. Age, availability of support (social and financial), and cancer type are factors that should be considered when assessing patients for unmet needs. There are currently few local support programs for patients with cancer to address these needs. This, in addition to the current emphasis on clinical care, indicates the need to urgently develop patient support programs that address unmet needs across the health system and information, psychological, and physical and daily living domains. Furthermore, it will be necessary to review current patient care pathways in many Nigerian cancer treatment centers and incorporate patient navigators who can sufficiently provide patients with necessary health information as needed.

Few limitations exist and have implications for the generalizability of the current study. Our study was carried out in one cancer treatment center in Nigeria. Although this is a major referral center for cancer cases in southwest Nigeria and other parts of the country, it does not fully incorporate all areas in Nigeria. In addition, findings in the sexuality need domain must be interpreted with caution, as the conservative nature of the Nigerian society may have introduced some bias into the responses, and the use of private interviews may help to increase the reliability of results obtained in the sexual domain of the questionnaire.

Nigerian patients with cancer experience unmet needs across many areas, especially in the health information and psychological domains. Hence, support and services that address these needs should be prioritized toward ensuring increased satisfaction with care, improved quality of life, and better treatment outcomes.
